# Safety of azithromycin in infants under six months of age in Niger: A community randomized trial

**DOI:** 10.1371/journal.pntd.0006950

**Published:** 2018-11-12

**Authors:** Catherine E. Oldenburg, Ahmed M. Arzika, Ramatou Maliki, Mohamed Salissou Kane, Elodie Lebas, Kathryn J. Ray, Catherine Cook, Sun Y. Cotter, Zhaoxia Zhou, Sheila K. West, Robin Bailey, Travis C. Porco, Jeremy D. Keenan, Thomas M. Lietman, MORDOR Study Group

**Affiliations:** 1 Francis I. Proctor Foundation, University of California, San Francisco, San Francisco, California, United States of America; 2 Department of Ophthalmology, University of California, San Francisco, San Francisco, California, United States of America; 3 Department of Epidemiology and Biostatistics, University of California, San Francisco, San Francisco, California, United States of America; 4 The Carter Center Niger, Niamey, Niger; 5 The Dana Center, Johns Hopkins University School of Medicine, Baltimore, Maryland, United States of America; 6 Department of Infectious and Tropical Diseases, London School of Hygiene and Tropical Medicine, London, United Kingdom; University of Oklahoma, UNITED STATES

## Abstract

**Background:**

Mass azithromycin distribution reduces under-5 child mortality. Trachoma control programs currently treat infants aged 6 months and older. Here, we report findings from an infant adverse event survey in 1–5 month olds who received azithromycin as part of a large community-randomized trial in Niger.

**Methods and principal findings:**

Active surveillance of infants aged 1–5 months at the time of treatment was conducted in 30 randomly selected communities from within a large cluster randomized trial of biannual mass azithromycin distribution compared to placebo to assess the potential impact on child mortality. We compared the distribution of adverse events reported after treatment among azithromycin-treated versus placebo-treated infants. From January 2015 to February 2018, the caregivers of 1,712 infants were surveyed. Approximately one-third of caregivers reported at least one adverse event (azithromycin: 29.6%, placebo: 34.3%, risk ratio [RR] 0.86, 95% confidence interval [CI] 0.68 to 1.10, *P* = 0.23). The most commonly reported adverse events included diarrhea (azithromycin: 19.3%, placebo: 28.1%, RR 0.68, 95% CI 0.49 to 0.96, *P* = 0.03), vomiting (azithromycin: 15.9%, placebo: 21.0%, RR 0.76, 95% CI 0.56 to 1.02, *P* = 0.07), and skin rash (azithromycin: 12.3%, placebo: 13.6%, RR 0.90, 95% CI 0.59 to 1.37, *P* = 0.63). No cases of infantile hypertrophic pyloric stenosis were reported.

**Conclusions:**

Azithromycin given to infants aged 1–5 months appeared to be safe. Inclusion of younger infants in larger azithromycin-based child mortality or trachoma control programs could be considered if deemed effective.

**Trial registration:**

ClinicalTrials.gov NCT02048007.

## Author summary

Trachoma control programs currently treat all adults and children age 6 months and older in communities endemic for trachoma. If shown to be safe, programs could consider inclusion of younger children in mass treatment programs. Here, we evaluated adverse events in infants aged 1–5 months who were participating in a placebo-controlled trial of mass azithromycin for the reduction of child mortality in Niger. Overall, there was no difference in the frequency of adverse events among children treated with azithromycin compared to placebo. Common adverse events in both arms included diarrhea, vomiting, and skin rash. Azithromycin distribution to children between 1 and 5 months of age appeared to be safe. Inclusion of younger children in azithromycin-based trachoma and child mortality programs could be considered.

## Introduction

Mass azithromycin distribution has been a core component of the World Health Organization (WHO)’s trachoma control program, with over 700 million doses of azithromycin distributed to adults and children aged 6 months and older [1,2]. Mass azithromycin distribution dramatically reduces the prevalence of the ocular strains of *Chlamydia trachomatis* that lead to trachoma [3–7]. For most indications, azithromycin is approved by the Federal Drug Administration (FDA) for use in children over 6 months of age, and programmatic treatment of children under 6 months with azithromycin has been limited by lack of safety data [8]. Observational studies have documented an increase in infantile hypertrophic pyloric stenosis (IHPS) following the use of macrolides in a child’s first month of life, with the greatest risk associated with macrolide using during the first 14 days and with erythromycin in particular [9–11]. These studies are limited by confounding by indication, as infants receiving macrolides are generally sicker than their untreated peers and may have different indications for treatment than those receiving other antibiotic classes. If shown to be safe, treatment of children less than 6 months of age may be beneficial for trachoma control programs, as infants infected with *C*. *trachomatis* have been shown to have a higher chlamydial load [4]. Higher chlamydial loads have been shown to correlate with disease severity and children with higher loads may be more likely to transmit infection [12].

Recently, the MORDOR (*Macrolides Oraux pour Réduire les Décès avec un Oeil sur la Résistance*) trial found a 14% reduction in all-cause child mortality following four rounds of biannual mass azithromycin distribution to all children aged 1–59 months in Malawi, Niger, and Tanzania compared to biannual placebo [13]. In MORDOR, the largest effects were seen in children under 6 months of age, with nearly 1 in 4 deaths averted in azithromycin-treated communities. MORDOR enrolled children as young as 1 month (>28 days) of age. The primary MORDOR trial was a large simple trial [14], and as such was not designed to efficiently evaluate adverse events other than mortality. Therefore, 30 communities were randomly selected from each country for more intensive monitoring, including active adverse event assessments. Here, we present adverse event data from children aged 1 to 5 months from the Niger site of the trial, to establish the safety of provision of azithromycin to infants under 6 months of age.

## Methods

### Trial setting and eligibility

MORDOR was a community randomized, placebo-controlled trial conducted in Mangochi, Malawi, Boboye and Loga, Niger, and Kilosa, Tanzania (ClinicalTrials.gov NCT02048007) that compared biannual azithromycin distribution compared to biannual placebo distribution for the prevention of childhood mortality. The current study is restricted to the Niger study site. Methods for the trial have been previously reported [13]. Eligible communities had a population between 200 and 2,000 inhabitants on the most recent national census. Children were eligible for treatment if they were between 1 and 59 months of age and weighed at least 3,800 grams at the time of treatment. In each country, 30 of the randomized communities (15 per arm) were randomly selected to participate in a morbidity sub-study. The morbidity communities included additional assessment of nutritional status as well as rectal swabs, nasal swabs, nasopharyngeal swabs, and dried blood spot collection in a random sample of 50 children in each community. The study intervention (biannual azithromycin or placebo) and census were identical in morbidity and mortality communities. The infant adverse event study was conducted in morbidity communities in Niger following each treatment round among all infants aged 1 to 5 months per the most recent census.

#### Ethics statement

This study was approved by the Committee on Human Research at the University of California, San Francisco (10–01036) and the Institutional Review Board at the Niger Ministry of Health (034/2017/CNERS). Due to low literacy levels in the study area, verbal informed consent was obtained from village leaders and guardians of children. Caregiver assent for their child to participate in the study was documented in the study mobile application. Both institutional review boards approved the consent procedure. The study was conducted in accordance with the Declaration of Helsinki.

### Randomization and masking

Morbidity communities were randomized in a 1:1 fashion using R (R Foundation for Statistical Computing, Vienna, Austria). Participants, observers, investigators, and those performing data cleaning were masked to treatment arm. The placebo was identical in appearance and in packaging to the oral azithromycin suspension.

### Census

A door-to-door census was conducted prior to each treatment round. All children aged 0–59 months and pregnant women were enumerated. Vital status was assessed (dead, alive, unknown) at each follow-up census. Children who were aged between 1 and 5 months during each census round in the morbidity communities were eligible for the infant adverse event survey.

### Intervention

Every child aged 1–59 months at the most recent census was offered a single dose of directly observed oral azithromycin or placebo (both provided by Pfizer, Inc, New York City). Each child was given a volume of suspension equivalent to 20 mg/kg as estimated by height stick approximation (per Niger’s trachoma guidelines) or by weight for those unable to stand. Treatment was given after each examination round, with a treatment coverage target of 80%.

### Adverse event survey

Following each treatment round, the caregivers of infants aged 1–5 months were interviewed regarding adverse events since the last treatment, with a goal of interviewing caregivers within 2 weeks of treatment. A list of all infants aged 1–5 months based on the most recent census in each morbidity community was generated, and we attempted to interview caregivers of all children. Caregivers were asked if their child was treated as part of the study, and for those treated, if the child had a health problem in the two-week period following treatment and if the child was brought to a health clinic for treatment. Only caregivers of children who received the study treatment were asked about health problems to estimate the incidence of health problems in treated children, as inclusion of untreated children could have biased estimates towards the null. To estimate the intention-to-treat effect, all caregivers, regardless of whether or not the mother reported that the child was treated, were asked if the child had any of the following symptoms since the last time the study team visited the child’s community: abdominal pain, vomiting, nausea, diarrhea, dyspepsia, constipation, hemorrhoids, or skin rash.

### Sample size considerations

The sample size for the morbidity communities was based off the primary morbidity outcome, which was macrolide resistance in *Streptococcus pneumoniae*. We assumed 12% baseline resistance (based on previous studies) and an ICC of approximately 0.051 (based on the Trachoma Elimination Follow-up study [15]). We estimated that inclusion of 30 villages (15 per arm) and 10 samples per community would yield approximately 80% power to detect a difference in prevalence of resistance of 18% (e.g., 12% versus 30%) assuming 80% carriage of *S*. *pneumoniae*. For the infant adverse event survey, the sample size was limited by the number of 1 to 5 month old children residing in the 30 communities during the study period.

### Statistical analysis

Descriptive characteristics were calculated with medians and interquartile ranges (IQR) for continuous variables and proportions for categorical variables. Generalized linear models were used to compare 1) if the child had a health issue within two weeks of treatment and 2) if the caregiver sought medical care for the child within two weeks of treatment between the azithromycin and placebo arms. Because the survey restricted questions related to health issues following treatment to children who had received treatment, models were restricted only to children who received treatment per caregiver report. A repeated measures model was used to assess whether there was an overall difference in the distribution of adverse events in azithromycin- versus placebo-treated infants, with a random effect for each child and study community. To estimate risk ratios for any adverse event and each adverse event individually in azithromycin-treated infants compared to placebo-treated infants, we used generalized linear models with a binomial distribution and log link, with standard errors clustered by the community of residence of the infant (the unit of randomization). All analyses were conducted in Stata version 14.2 (StataCorp, College Station, TX) and R version 3.4.3 (The R Foundation for Statistical Computing).

## Results

**Fig 1:**
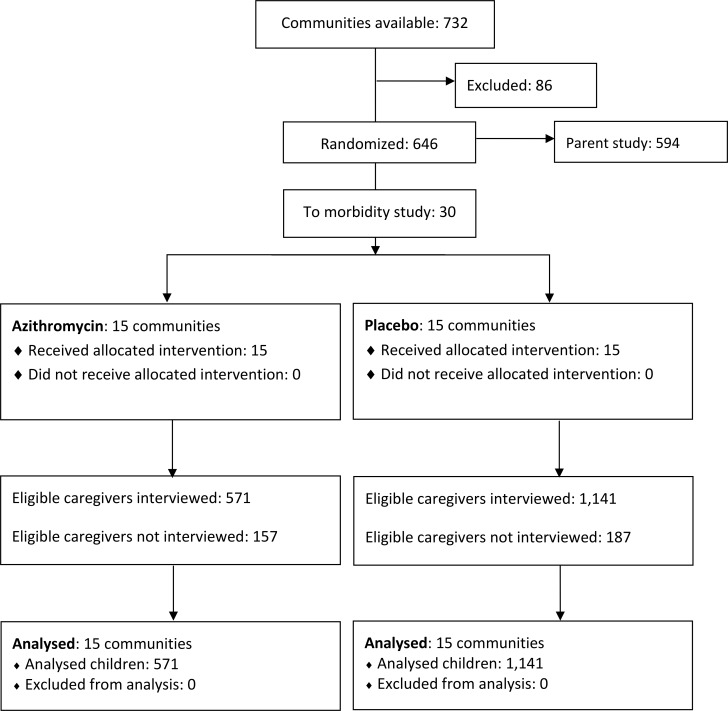
CONSORT diagram for the trial.

Of 2,056 eligible infants, caregivers of 1,712 (83.3%) were interviewed between June 2015 and February 2018 (Fig 1). The median time between each community’s treatment and the caregiver survey was 34 days (IQR 21 to 61 days). There was no difference in the time between treatment and caregiver survey between azithromycin and placebo-treated communities (*P* = 0.78). Table 1 shows baseline characteristics of communities and infants included in this study. Approximately half of the children were female (48.5%) and median age at the time of the census was 2 months (IQR 1 to 4 months). Caregivers reported that 70.2% (*N* = 1,201) of infants received study treatment, with no differences in treatment between arms (*P* = 0.22).

**Table 1:** Characteristics by study arm.

	Azithromycin	Placebo
Caregiver interviews	N = 571	N = 1,141
Female, N (%)	280 (49.7%)	542 (47.9%)
Age at survey, months (median, IQR)	2 (1 to 4)	3 (1 to 4)

Abbreviations: IQR, interquartile range

Among infants for whom the caregiver reported receiving study treatment, there was no difference in reports of any health problems in the two-week period following treatment (34.1% azithromycin versus 40.3% placebo, *P* = 0.24, Table 2). Similarly, there was no difference in visiting a health clinic for a health problem among those who received treatment (*P* = 0.27).

**Table 2:** Healthcare visits following treatment among infants aged 1–5 months receiving treatment (N = 1,201).

	Azithromycin	Placebo	RR (95% CI)	*P*-value
Any health problem	131 (34.1%)	329 (40.3%)	0.85 (0.64 to 1.12)	0.24
Had health problem and visited clinic	49 (12.8%)	137 (16.8%)	0.76 (0.47 to 1.23)	0.27

There was no difference in the overall distribution of adverse events in children between the two study arms (*P* = 0.43, repeated measures model). Overall, caregivers of 32.7% of all infants reported at least one adverse event (Table 3). The most commonly reported adverse events in the period following treatment included diarrhea (25.2%), vomiting (19.3%), and skin rash (13.1%). There was no difference overall in report of any adverse event (RR 0.86, 95% CI 0.68 to 1.10, *P* = 0.23). Infants in the azithromycin-treated arm had reduced risk of diarrhea (RR 0.68, 95% CI 0.49 to 0.96, *P* = 0.03) and hemorrhoids (RR 0.27, 95% CI 0.08 to 0.87, *P* = 0.03). The distribution of all other adverse events was similar between treatment arms. There were no reported cases of IHPS.

**Table 3:** Adverse events following azithromycin treatment among infants aged 1–5 months in communities randomized to azithromycin or placebo (N = 1,712).

	Azithromycin	Placebo	RR (95% CI)	*P*-value
Any adverse event	169 (29.6%)	391 (34.3%)	0.86 (0.68 to 1.10)	0.23
Abdominal pain^1^	52 (9.1%)	116 (10.2%)	0.90 (0.45 to 1.77)	0.75
Vomiting^1^	91 (15.9%)	240 (21.0%)	0.76 (0.56 to 1.02)	0.07
Nausea^1^	28 (4.9%)	55 (4.8%)	1.02 (0.34 to 3.04)	0.98
Diarrhea^1^	110 (19.3%)	321 (28.1%)	0.68 (0.49 to 0.96)	0.03
Dyspepsia^1^	17 (3.0%)	20 (1.8%)	1.70 (0.25 to 11.45)	0.59
Constipation^1^	32 (5.6%)	66 (5.8%)	0.97 (0.42 to 2.23)	0.94
Hemorrhoids^1^	7 (1.2%)	52 (4.6%)	0.27 (0.08 to 0.87)	0.03
Skin rash^1^	70 (12.3%)	155 (13.6%)	0.90 (0.59 to 1.37)	0.63

^1^Assessed via caregiver report, overall *P* = 0.43

## Discussion

Trachoma control programs currently only treat children aged 6 months and older, with younger children receiving topical tetracycline. The majority of FDA-approved indications for azithromycin include children aged 6 months and older. Here, we were unable to find any evidence of an increase in adverse events in a sample of infants receiving azithromycin versus placebo as part of a community randomized trial. These results suggest that azithromycin treatment may be safe in infants under 6 months of age, and expansion of indications for azithromycin in public health programs such as trachoma control to include children under 6 months of age could be considered.

Previous evaluation of the safety of azithromycin in children under 6 months of age have consisted of large epidemiologic studies or small randomized controlled trials of azithromycin use among very low birth weight neonates for the prevention of bronchopulmonary dysplasia [8–10,16–19]. Epidemiologic cohorts have shown no increase in risk of IHPS in children over 6 weeks of age compared to untreated children, but may be subject to confounding by indication. In the general population, the vast majority of IHPS cases are diagnosed during the first 12 weeks of life, with a sharp decline in incidence after the 5-6^th^ week of life [20]. Population-based estimates of IHPS in sub-Saharan Africa are rare, but IHPS is thought to be less common in sub-Saharan Africa than in other regions, potentially due to differences in practices that have been shown to increase risk, such as bottle and formula feeding [21–23]. Although no cases of IHPS were reported, the present study was underpowered to assess IHPS, given its rarity particularly among children over one month of age [9,20]. Projectile vomiting is the most common symptom of IHPS [24], however the risk of vomiting in the present study was lower in azithromycin-treated infants compared to placebo-treated infants, suggesting that azithromycin did not lead to IHPS in this population. While future studies using azithromycin in children under 12 weeks of age should remain vigilant in screening for IHPS, the results of this study suggest that the risk of IHPS is likely rare in this population.

Trachoma control programs distribute azithromycin to children and adults aged 6 months and older in communities with endemic trachoma. Although infants under 6 months are thought to have lower infection rates than older children [25], earlier treatment of infants for trachoma may reduce community prevalence of *C*. *trachomatis* as infants may have a higher chlamydial load if infected [4]. Caregivers of infants under 6 months of age are given topical tetracycline ointment with instructions to apply the ointment daily for 6 weeks, however completion of the regimen is generally thought to be poor [25–28]. The ability to expand azithromycin distribution to children as young as one month of age could potentially contribute to reductions in trachoma in endemic regions if treatment of children under 6 months of age with azithromycin is shown to be effective for trachoma control.

A subgroup analysis of the MORDOR study demonstrated a nearly 25% reduction in mortality among infants under 6 months of age compared to an overall decrease of 14%, generating the hypothesis that the largest effects of azithromycin for prevention of child mortality may be in the youngest age groups [13]. Previous studies have shown a significant decrease in child mortality among children aged 6–59 months in the context of azithromycin distribution for trachoma control [29–31]. Younger children are at higher risk of mortality compared to older children [32]. The potential for benefit from a mortality-reducing intervention, such as azithromycin, may be greater in this age group than in all children under the age of 5. In the parent study, which included more than 300,000 person-years at risk, few adverse events were reported, although active surveillance was not undertaken. The parent study was a large simple trial designed specifically to evaluate the effect of azithromycin on mortality, which can be considered the most serious adverse event. However, this design was not efficient for evaluation of other adverse events due to the sample size required for the study to be adequately powered for the mortality outcome, given that mortality is a rare event. Active adverse event monitoring was therefore only conducted in the smaller morbidity study, which included more intensive monitoring of study participants.

The results of this study must be considered in the context of several limitations. MORDOR did not enroll children under one month of age, and thus we cannot comment on the safety of azithromycin in neonates. Active surveillance was conducted via caregiver report, which could be subject to social desirability or recall biases. Due to inability to link study records to clinic records, we did not attempt to validate caregiver responses against health post or hospital records. Estimates may therefore be an over- or underestimate of the true burden of adverse events. Although we planned to survey caregivers within two weeks of treatment, due to logistical challenges the survey was often conducted several weeks following treatment. A longer duration between treatment and the survey could increase the likelihood of misclassification. However, due to the use of masked placebo, any misreporting is unlikely to be differential with respect to study arm. In addition, a longer duration between treatment and the survey could increase the number of events that occurred with decreased probability that they were related to study treatment, which could bias results towards the null. However, there were no significant differences in the effect of azithromycin versus placebo by timing of the survey on any adverse event reporter in this study. Although we attempted to interview the caregiver of each child, only 83% of eligible children’s caregivers were interviewed. Due to the placebo-masked nature of the study, differential response by arm is unlikely, however it is possible that caregivers of children who died were less likely to be interviewed. The probability of mortality is very low, and thus unlikely to substantially bias results. This study was conducted in one of three of the MORDOR trial sites, in a region of the Sahel with very high child mortality and infection rates. The results of this study may only be generalizable to other regions with similar distributions of childhood infection.

The results of MORDOR suggest there may be a large reduction in mortality in the 1 to 5 month age group with the use of azithromycin, but as a large simple trial, the trial was not ideal for quantifying common adverse events. In this ancillary study, we were unable to find a difference in adverse events in infants aged 1 to 5 months participating in a large community-randomized trial of biannual mass azithromycin distribution for prevention of child mortality. Currently, several guidelines indicate azithromycin for use in children over 6 months of age. These results suggest that azithromycin could be considered in infants over 1 month of age, and their inclusion in various public health programs using azithromycin may be appropriate.

## Supporting information

S1 Appendix MORDOR group authorship list. (DOCX)

S2 Appendix CONSORT 2010 checklist. (DOC)
