# Supplementary material for: Safety of azithromycin in infants under six months of age in Niger: A community randomized trial
Source: PLoS Negl Trop Dis. 2018 Nov 12;12(11):e0006950. doi: 10.1371/journal.pntd.0006950 (PMC6258425; doi:10.1371/journal.pntd.0006950)
Supplement: S1 Appendix — (DOCX) [file pntd.0006950.s001.docx]

*University of California, San Francisco, San Francisco, CA, USA* – Catherine A Cook, Sun Y Cotter, Thuy Doan, Dionna M Fry, Bruce D Gaynor, Jeremy D Keenan, Elodie Lebas, Thomas M Lietman, Kieran S O’Brien, Catherine E Oldenburg, Travis C Porco, Kathryn J Ray, Philip J Rosenthal, George W Rutherford, Nicole E Stoller, Benjamin Vanderschelden, John P Whitcher, Zhaoxia Zhou, Lina Zhong; *London School of Hygiene and Tropical Medicine, London, UK* – Robin L Bailey, Sarah E Burr, John Hart, David CW Mabey, Anthony W Solomon; *Johns Hopkins University, Baltimore, MD, USA* – Evan M Bloch, Christian L Coles, Kurt Dreger, Hemjot Kaur, Alain B Labrique, Beatriz Munoz, Alfred Sommer, Jerusha Weaver, Sheila K West; *Blantyre Institute for Community Ophthalmology, Blantyre, Malawi* – Alvin Chisambi, Khumbo Kalua, Zachariah Kamwendo; *University of Malawi College of Medicine, Blantyre, Malawi* – Ken Maleta; *The Carter Center, Atlanta, GA, USA* – E Kelly Callahan, Aisha E Stewart; *The Carter Center Niger, Niamey, Niger* – Ahmed M Arzika, Abdou Moumouni Goundara, Salissou Kane, Ramatou Maliki; *Programme National de Santé Oculaire, Niamey, Niger* – Amza Abdou, Nassirou Beido, Boubacar Kadri; *Muhimbili University of Health and Allied Sciences, Dar es Salaam, Tanzania* – Mabula Kasubi; *National Institute for Medical Research, Dar es Salaam, Tanzania* – Leonard Mboera, Zakayo Mrango; *International Trachoma Initiative, Decatur, GA, USA*- Paul M Emerson, Huub Gelderbloom, MD, PJ Hooper.

*Steering Committee* – Robin L Bailey, Jeremy D Keenan, Thomas M Lietman (PI), Travis C Porco, and Sheila K West.

*Data and Safety Monitoring Committee: University of Washington, Seattle, WA, USA –* Judd L Walson*; Liverpool School of Tropical Medicine, Liverpool, UK –* Allen W Hightower*; Loyola University, Chicago, IL, USA –* Emily E Anderson*, Berhan Public Health & Eye Care Consultancy, Addis Ababa, Ethiopia –* Wondu Alemayehu*; Tulane University, New Orleans, LA, USA –* Latha Rajan*.*

*Bill & Melinda Gates Foundation, Seattle, WA, USA* – Rasa Izadnegahdar, Julie Jacobson, Thomas Kanyok, Erin Shutes.

*Pfizer, New York, NY, USA* –Julie Jensen, Chuck Knirsch, John Schenkel
